# Comparison of adverse events associated with different spacers used with non-extrafine beclometasone dipropionate for asthma

**DOI:** 10.1038/s41533-019-0115-0

**Published:** 2019-02-08

**Authors:** Simon Wan Yau Ming, John Haughney, Dermot Ryan, Shishir Patel, Matthias Ochel, Martina Stagno d’Alcontres, Susannah Thornhill, Janwillem W. H. Kocks, David Price

**Affiliations:** 1 Observational and Pragmatic Research Institute, Singapore, Singapore; 2 University of Aberdeen, Aberdeen, UK; 3 Optimum Patient Care, Cambridge, UK; 4 University of Edinburgh, Edinburgh, UK; 5 Chiesi Ltd, Manchester, UK; 6 General Practitioners Research Institute, Groningen, The Netherlands

## Abstract

Co-prescription of Aerochamber^®^ spacer with non-extrafine beclometasone diproprionate (non-EF BDP) is common but unlicensed. We report a comparison of inhaled corticosteroid (ICS)-related adverse events between patients co-prescribed Aerochamber compared to the licensed Volumatic^®^ spacer. We utilised two historical cohorts: questionnaire-based and electronic medical record (EMR)-based, to assess patient-reported and EMR-recorded adverse events in patients with asthma prescribed non-EF BDP. Marginal effect estimate (MEE) was calculated to determine non-inferiority of Aerochamber compared to Volumatic in terms of patient-reported oral thrush and hoarseness with margin of 0.13. Other patient-reported adverse events (sore throat, bruising, weight gain, and coughing), and EMR-recorded adverse events were also assessed. Rate of patient-reported oral adverse events were non-inferior in 385 patients prescribed Aerochamber compared to 155 patients prescribed Volumatic (27.7 vs 29.9%; MEE, −0.043; 95% CI, −0.133 to 0.047). Total patient-reported adverse events did not differ significantly between Aerochamber and Volumatic (53.3 vs 49.7% with ≥1 adverse event). The EMR-based study of 1471 matched pairs of subjects did not show significantly different number of EMR-recorded adverse events between Aerochamber and Volumatic (12.5 vs 12.8% with ≥1 adverse events). Co-prescribing Aerochamber with non-EF BDP does not increase the risk for patient-reported and EMR-recorded ICS-related adverse events compared to co-prescribing Volumatic.

## Asthma: checking up on spacer safety

A study of spacer devices for use with asthma inhalers shows no significant difference in the incidence of side-effects between licensed and unlicensed spacers. Spacers offer a way of improving the dose inhaled, particularly in children and those adults who struggle to use asthma inhalers correctly. However, non-extrafine beclomethasone dipropionate (like Clenil Modulite) inhalers are frequently co-prescribed with unlicensed Aerochamber spacers rather than the Volumatic spacer which is licensed for this drug. David Price at the University of Aberdeen, UK, and co-workers analysed data from two historical studies to verify whether Aerochamber use enhanced the risk of unpleasant side effects for patients, such as oral thrush or hoarseness. They found no significant difference between licensed and unlicensed spacers and the chances of adverse events, suggesting both are suitable for use with non-extrafine beclomethasone dipropionate inhalers.

## Introduction

Asthma is a heterogeneous disease characterised by chronic airway inflammation that has a substantial impact on quality of life and healthcare resources. National and international guidelines recommend inhaled corticosteroids (ICS) as the first-line therapy for treatment of asthma.^1,2^ ICS treatment has proven to be efficient at improving lung function, decreasing airway hyperresponsiveness, reducing symptoms, frequency, and severity of exacerbations, and improving patient quality of life.^3–5^ Despite their proven efficacy, ICS can cause both oropharyngeal and systemic adverse events.^6–10^

Oropharyngeal adverse events associated with ICS use include oral candidiasis (oral thrush), hoarseness, dysphonia, pharyngitis, and cough reflex.^11–13^ Oral thrush is a well-documented adverse event associated with regular use of ICS in patients with asthma.^6,11,14,15^ Approximately 5–10% of patients prescribed ICS reported adverse events in the oral cavity and pharynx,^7,11,12^ with the occurrence of clinically significant oropharyngeal candidiasis as high as 10% in adults^6,16,17^ and between 1 and 3% in children.^18,19^ The reduction of the local immune response,^20^ or growth stimulation of *Candida albicans*^21^ through an increase in salivary glucose, are believed to be responsible for the development of candidiasis. Multiple factors have been reported to contribute to the incidence of oral thrush in patients with asthma, including the type and dose of ICS prescribed, the delivery device used, and patient adherence to medication instructions.^22–24^ This relationship between risk of oral thrush and the type, dose, and delivery device of ICS has also been observed in chronic obstructive pulmonary disorder (COPD) patients.^6^

The use of spacers with pressurised metered dose inhalers (pMDIs)^25^ and careful mouth rinsing after using dry powder inhalers can reduce the risk of oral thrush.^18^ Spacers are recommended by asthma treatment guidelines for patients under the age of 16 years, for those who have problems coordinating actuation, for those prescribed high-dose ICS, for those at risk of suffering from local side effects, and for elderly patients.^26^ The addition of a spacer to a pMDI has proven to consistently reduce aerosol velocity and particle size in the aerosol plume, thus reducing the amount of prescribed therapy deposited in the oropharyngeal cavity and increasing the amount of active compound that reaches the lung.^27–29^ Previous studies have suggested that spacers used with non-extrafine (non-EF) particle ICS may result in reduced rates of oropharyngeal candidiasis.^28,30,31^

Spacers are licensed for use with specific inhalers. Non-EF beclometasone dipropionate (BDP) (Clenil^®^ Modulite^®^) is licensed for use only with the Volumatic^®^ spacer.^32^ However, our previous study found that the Aerochamber^®^ spacer has also been frequently prescribed, off-label, in conjunction with Clenil Modulite.^33^ There is concern that the use of unlicensed spacers may result in a greater number of adverse events compared to the use of licensed spacers. Large spacers, such as the Volumatic device, have been shown to have more effective drug distribution compared to smaller spacers, such as the Aerochamber.^34,35^ However, the ease of use and carriage of the smaller Aerochamber device make it a more attractive choice for both patients and prescribers, even if it is not licensed.^36^ The aim of this study was to characterise both patient-perceived and electronic medical record (EMR)-recorded possible ICS-related adverse events in patients with asthma co-prescribed the licensed Volumatic or the unlicensed Aerochamber spacer with their non-EF BDP therapy. This was conducted using two historical cohort studies: a questionnaire-based study and an EMR-based study for assessment of patient-reported and EMR-recorded adverse events, respectively.

## Results

### Study population

**Fig. 1:**
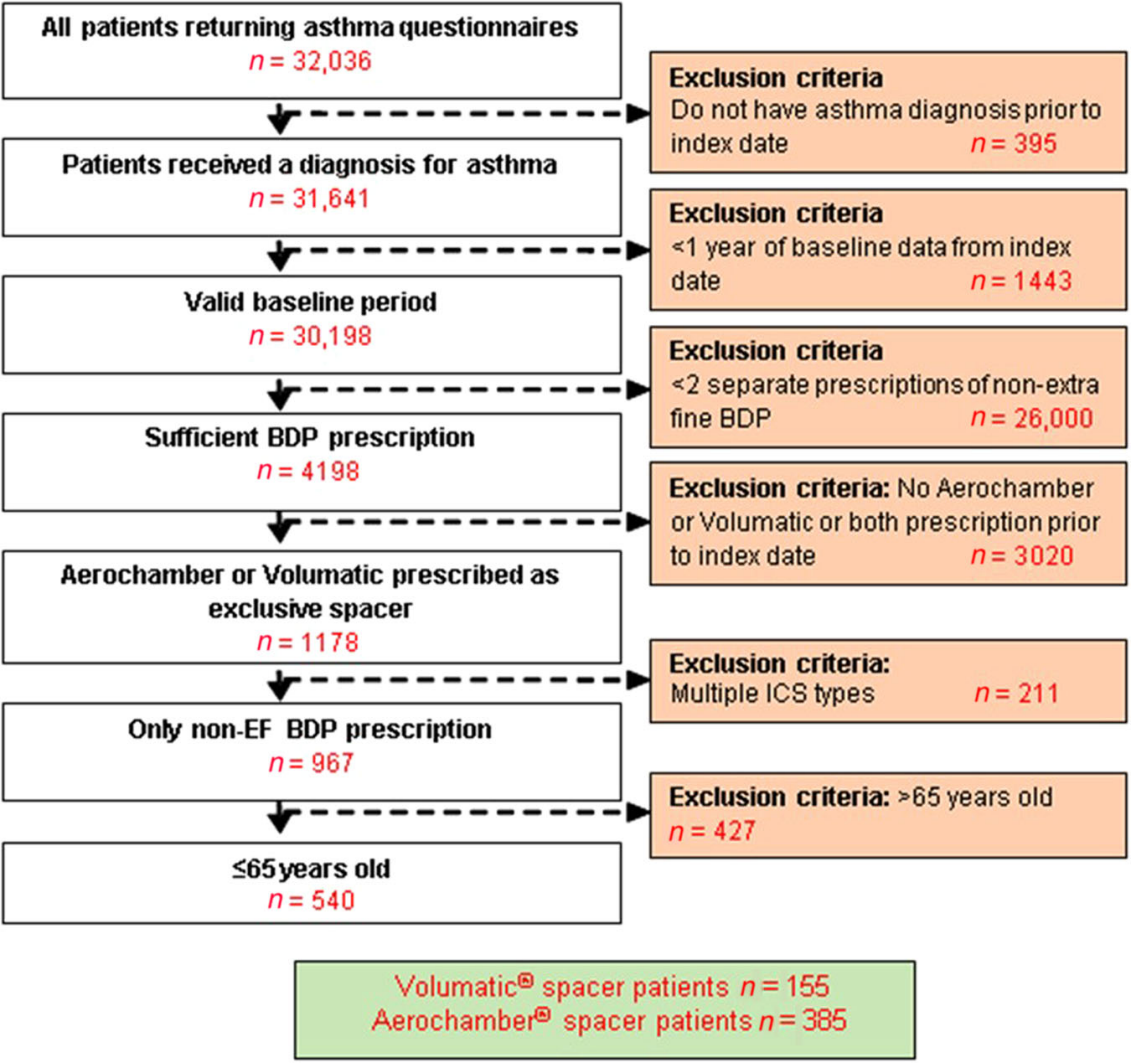
Patient flow diagram for the questionnaire-based study (primary objective)

The questionnaire-based study consisted of 540 patients who had questionnaire data, collected for routine practice purpose, of whom 385 were prescribed the Aerochamber spacer and 155 were prescribed the Volumatic (Fig. 1). The group prescribed Aerochamber had significantly more female patients (65.7 vs 54.8%) and more current smokers (27.2 vs 14.1%) but were prescribed lower short-acting β2 agonist (SABA) average daily dosage at baseline (*p* = 0.003) (Table 1) compared to the Volumatic group.

**Table 1:** Baseline patient characteristics of questionnaire-based study

Baseline variable	Volumatic^®^ (*n* = 155)	Aerochamber^®^ (*N* = 385)	*P* value	RCC
Male gender, *n* (%)	70 (45.2)	132 (34.3)	0.0181	2.1
Age (completed years)				
Mean (SD)	42.4 (19.2)	46.7 (15.7)	0.0630	2.9
Median (IQR)	48.0 (32.0)	51.0 (20.0)		
Smoking status				
Non-smoker, *n* (%)	108 (72.5)	233 (62.6)	0.0058	0.2
Ex-smoker, *n* (%)	20 (13.4)	38 (10.2)		
Current smoker, *n* (%)	21 (14.1)	101 (27.2)		
Total non-missing (%)^a^	149 (96.1)	372 (96.6)		
BMI (kg/m²)				
<18.5, *n* (%)	15 (9.9)	26 (7.0)	0.4863	0.9
<18.5–24.99, *n* (%)	53 (35.1)	118 (32.0)		
25–29.99, *n* (%)	43 (28.5)	108 (29.3)		
≥30, *n* (%)	40 (26.5)	117 (31.7)		
Total non-missing (%)^a^	151 (97.4)	369 (95.8)		
SABA average daily dosage (µg)				
<100, *n* (%)	26 (16.8)	110 (28.6)	0.0026	2.4
100–200, *n* (%)	44 (28.4)	109 (28.3)		
201–400, *n* (%)	48 (31.0)	116 (30.1)		
>400, *n* (%)	37 (23.9)	50 (13.0)		
ICS average daily prescription (µg BDP equivalent)				
<100, *n* (%)	11 (7.1)	11 (2.9)	0.1447	1.9
100–250, *n* (%)	71 (45.8)	174 (45.2)		
251–500, *n* (%)	48 (31.0)	131 (34.0)		
>500, *n* (%)	25 (16.1)	69 (17.9)		
LABA ≥1 prescription, *n* (%)	14 (9.0)	41 (10.6)	0.5741	0.3
LTRA ≥1 prescription, *n* (%)	7 (4.5)	11 (2.9)	0.3313	0.4
Eczema diagnosis (1-year baseline), *n* (%)	69 (44.5)	139 (36.1)	0.0692	1.0
Rhinitis diagnosis (1-year baseline), *n* (%)	51 (32.9)	120 (31.2)	0.6951	0.2
Thrush diagnosis (1-year baseline), *n* (%)	11 (7.1)	20 (5.2)	0.3901	0.2
Percentage predicted peak flow				
>=80%, *n* (%)	77 (53.5)	177 (49.6)	0.3037	1.2
50–80%, *n* (%)	59 (41.0)	168 (47.1)		
<=50%, *n* (%)	8 (5.6)	12 (3.4)		
Total non-missing (%)^a^	144 (92.9)	357 (92.7)		
Severe asthma exacerbations in 1-year baseline^b^				
0, *n* (%)	129 (83.2)	321 (83.4)	0.3908	0.0
1, *n* (%)	22 (14.2)	45 (11.7)		
2, *n* (%)	2 (1.3)	15 (3.9)		
≥ 3, *n* (%)	2 (1.3)	4 (1.0)		

The *p* values were computed from chi-squared test for categorical variables, or Mann–Whitney test for continuous variables and variables presented as both continuous and categorical. Patients were not matched to preserve statistical power. Summary statistics are presented as counts and percentages unless stated otherwise. RCC indicating bias potential of variable when added into the model predicting the outcome

*RCC* relative change coefficient, *IQR* interquartile range, *BMI* body mass index, *SABA* short-acting β2 agonist, *ICS* inhaled corticosteroid, *BPD* beclometasone dipropionate, *LABA* long-acting β2 agonist, *LTRA* leukotriene receptor antagonist

^a^Missing data present for this variable, percentages for categorical variables are given as a percentage of the non-missing observations (out of 155 for Volumatic and 385 for Aerochamber)

^b^Defined as occurrence of either: (1) asthma-related unscheduled hospitalisation/accident & emergency (A&E) attendance, (2) an acute course of oral steroids, or (3) antibiotics prescribed with lower respiratory consultation

**Fig. 2:**
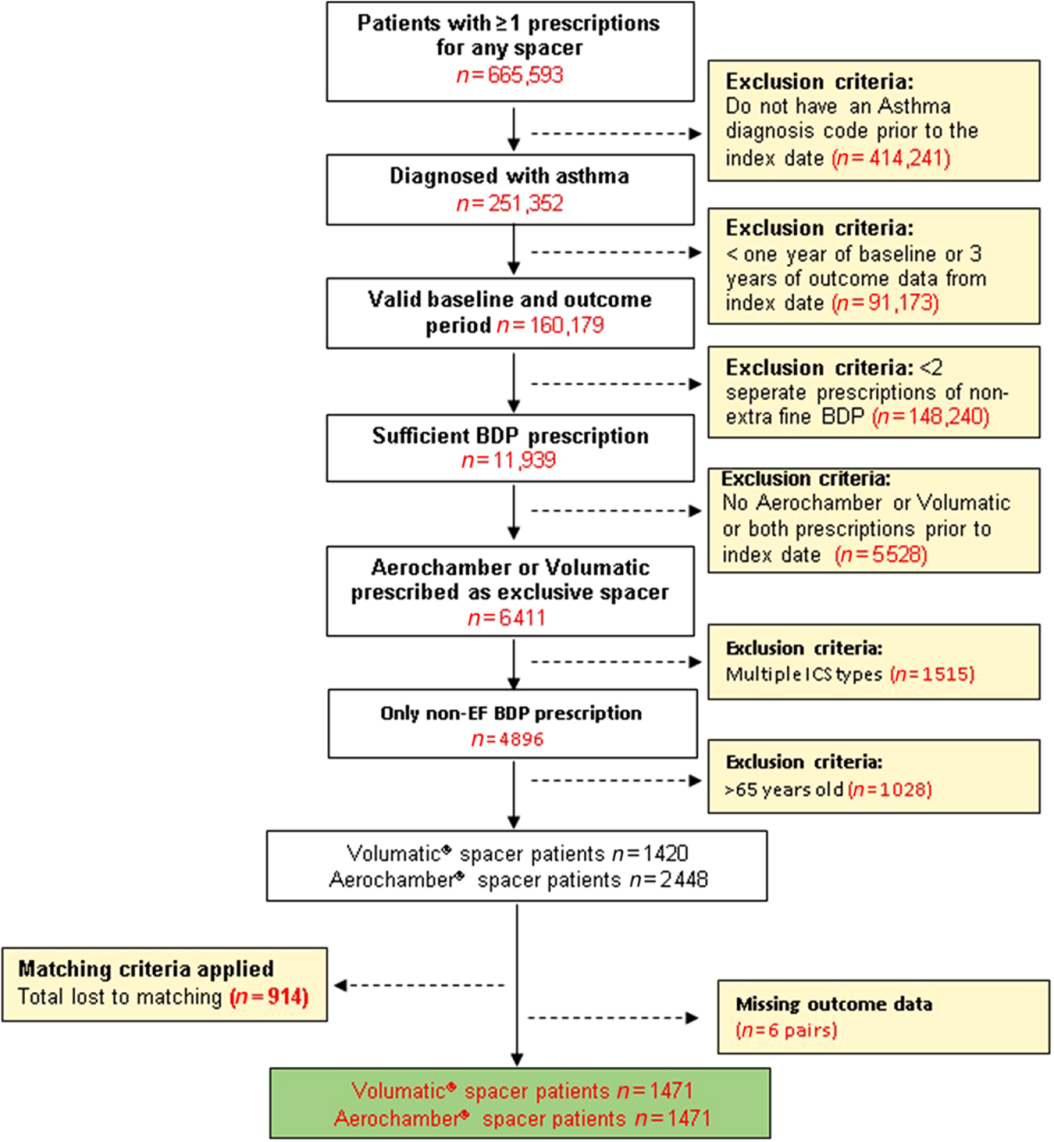
Patient flow diagram for the electronic medical record (EMR)-based study (secondary objective)

A total of 1471 matched pairs were included in the EMR-based study after 1:1 matching (Fig. 2) with the mean age (SD) of 30 (28.2) years and 54% patients were female (Table 2). More patients in the Aerochamber group were current smokers (17.9 vs 16.0%). Patients prescribed the Aerochamber spacer had a higher percentage predicted peak flow than those prescribed the Volumatic spacer (59.0 vs 52.5 with ≥80% predicted peak flow respectively, *p* = 0.006). However, the number of patients who experienced at least one severe asthma exacerbation during the 1-year baseline period was not significantly different between the Aerochamber- and the Volumatic-prescribed groups (24.5 vs 25.6 respectively, *p* = 0.650).

**Table 2:** Matched baseline patient characteristics of EMR-based study

Baseline variable	Volumatic^®^ (*n* = 1471)	Aerochamber^®^ (*N* = 1471)	*P* value
Male gender^a^, *n* (%)	678 (46.1)	678 (46.1)	1.0000
Age (completed years)^a^			
Mean (SD)	30.0 (28.2)	30.0 (28.2)	0.9079
Median (IQR)	11.0 (52.0)	11.0 (53.0)	
Smoking status			
Non-smoker, *n* (%)	1058 (78.8)	980 (74.4)	0.0064
Ex-smoker, *n* (%)	69 (5.1)	102 (7.7)	
Current smoker, *n* (%)	215 (16.0)	236 (17.9)	
Total non-missing (%)^b^	1342 (91.2)	1318 (89.6)	
BMI (kg/m²)			
<18.5, *n* (%)	350 (37.4)	363 (38.1)	0.5190
18.5−24.99, *n* (%)	121 (12.9)	129 (13.6)	
25–29.99, *n* (%)	122 (13.0)	103 (10.8)	
≥30, *n* (%)	342 (36.6)	357 (37.5)	
Total non-missing (%)^b^	935 (63.6)	952 (64.7)	
SABA average daily dosage (µg)			
<100, *n* (%)	216 (14.7)	210 (14.3)	0.5738
100–200, *n* (%)	374 (25.4)	393 (26.7)	
201–400, *n* (%)	514 (34.9)	530 (36.0)	
>400, *n* (%)	367 (24.9)	338 (23.0)	
ICS average daily prescription (µg BDP equivalent)^a^			
<100, *n* (%)	208 (14.1)	208 (14.1)	1.0000
100-250, n (%)	617 (41.9)	617 (41.9)	
251–500, *n* (%)	351 (23.9)	351 (23.9)	
>500, *n* (%)	295 (20.1)	295 (20.1)	
LABA ≥1 prescription, *n* (%)	215 (14.6)	223 (15.2)	0.6786
LTRA ≥1 prescription, *n* (%)	111 (7.5)	122 (8.3)	0.4527
Eczema diagnosis (1-year baseline), *n* (%)	669 (45.5)	613 (41.7)	0.0373
Rhinitis diagnosis (1-year baseline), *n* (%)	296 (20.1)	320 (21.8)	0.2768
Thrush diagnosis (1-year baseline), *n* (%)	77 (5.2)	70 (4.8)	0.5536
Percentage predicted peak flow			
>=80%	576 (52.5)	641 (59.0)	0.0064
50–80%	447 (40.7)	372 (34.2)	
<=50%	72 (6.6)	74 (6.8)	
Total non-missing (%)^b^	1099 (74.8)	1,087 (73.9)	
Severe asthma exacerbations in 1-year baseline^c^			
0, *n* (%)	1095 (74.4)	1111 (75.5)	0.6499
1, *n* (%)	245 (16.7)	248 (16.9)	
2, *n* (%)	83 (5.6)	72 (4.9)	
≥3, *n* (%)	48 (3.3)	40 (2.7)	

The *p* values were computed from chi-squared test for categorical variables, or Mann–Whitney test for continuous variables and variables presented as both continuous and categorical. Summary statistics were presented as counts and percentages unless stated otherwise

*EMR* electronic medical record, *IQR* interquartile range, *BMI* body mass index, *SABA* short-acting β2 agonist, *ICS* inhaled corticosteroid, *BPD* beclometasone dipropionate, *LABA* long-acting β2 agonist, *LTRA* leukotriene receptor antagonist

^a^Matching variables

^b^Missing data present for this variable, percentages for categorical variables are given as a percentage of the non-missing observations (out of 1471 patients in both groups)

^c^Defined as occurrence of either: (1) asthma-related unscheduled hospitalisation/accident & emergency (A&E) attendance, (2) an acute course of oral steroids, or (3) antibiotics prescribed with lower respiratory consultation

### Patient-reported oral adverse events

**Fig. 3:**
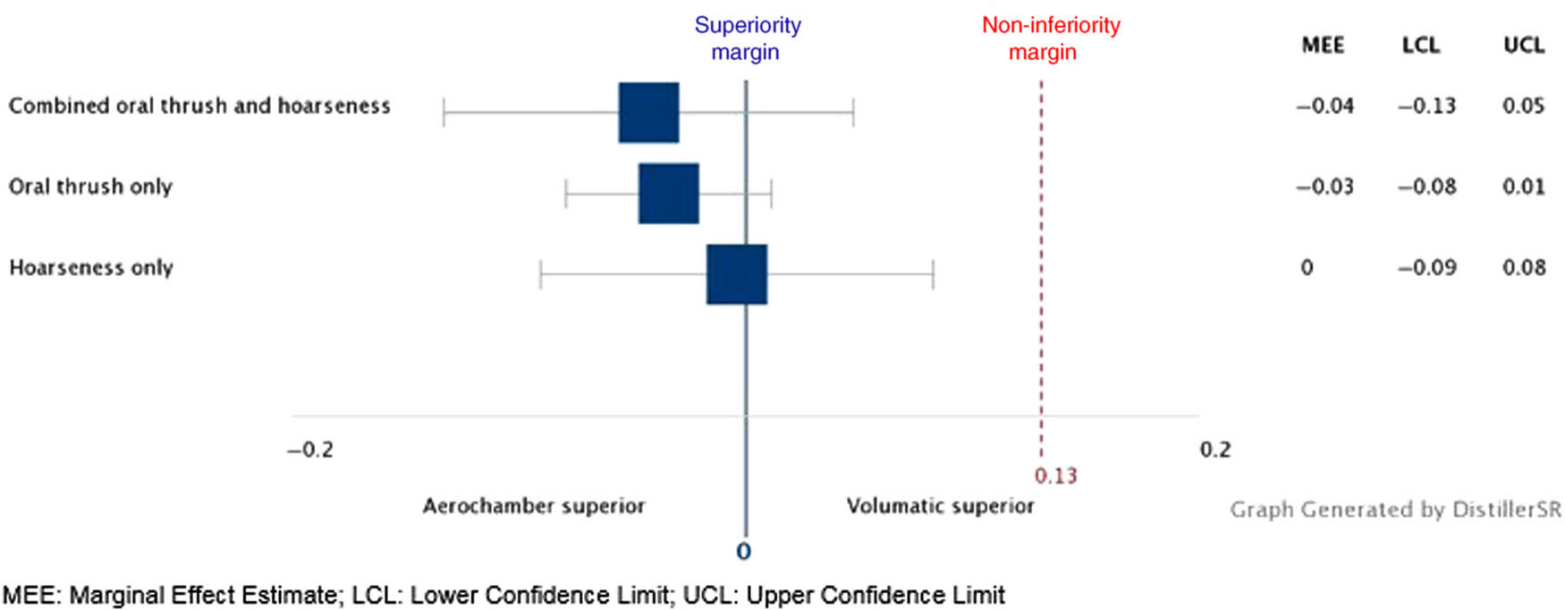
Non-inferiority of Aerochamber^®^ compared to Volumatic^®^ in patient-reported oral adverse events occurrence. Box indicates marginal effect estimate (MEE) value and whiskers indicate confidence intervals

Patient-reported oral adverse events (oral thrush or hoarse voice) were reported in 27.7% patients co-prescribed the unlicensed Aerochamber compared to 29.9% of patients co-prescribed the licensed Volumatic spacer. The marginal effect estimate (MEE) was −0.043 (95% confidence interval (CI), −0.133 to 0.047). As the upper limit of the 95% CI was less than the pre-defined non-inferiority margin of 0.13, the Aerochamber was determined to be non-inferior to the Volumatic spacer in terms of local oral adverse events (Fig. 3). Non-inferiority was also observed for the outcomes of oral thrush only (MEE, −0.034; 95% CI, −0.079 to 0.011) and hoarseness only (MEE, −0.004; 95% CI, −0.091 to 0.083).

### Total patient-reported adverse events

There were no significant differences in the total number of overall patient-reported adverse events (sore mouth/throat, bruising, abnormal weight gain, and cough in addition to oral thrush and hoarseness) between patients co-prescribed Aerochamber and patients co-prescribed Volumatic spacer (53.3 vs 49.7% with ≥1 reported event respectively, *p* = 0.797) from the questionnaire-based study.

### EMR-recorded adverse events

Of the 1471 patients in both groups prescribed Aerochamber and Volumatic, 1287 (87.5) and 1283 (87.2%) patients did not have any EMR-recorded adverse events, respectively. The number of patients with exactly one recorded adverse event were 169 (11.5) and 171 (11.6%), respectively, and 15 (1.0) and 17 (1.2%) had 2 or more adverse events in the Aerochamber and Volumatic groups, respectively (chi-square *p*-value = 0.931, Table 3).

**Table 3:** Number of EMR-recorded adverse events in matched patients aged 65 years or under

Number of adverse events, *n* (%)	0	1	2+	*P* value
Spacer device (*n* = 1471 each arm)				
Volumatic	1283 (87.2)	171 (11.6)	17 (1.2)	0.931
Aerochamber	1287 (87.5)	169 (11.5)	15 (1.0)	

Adverse events include: oral thrush, adrenal suppression diagnosis, osteoporosis/osteopenia, anxiety/depression, cataracts, and glaucoma

*EMR* electronic medical record

Analysed as a continuous variable (counts of adverse events), the rates of EMR-recorded adverse events were also not significantly different in patients prescribed Aerochamber compared to patients prescribed Volumatic spacer (adjusted rate ratio, 1.28; 95% CI, 0.99 to 1.65).

## Discussion

This real-life study in a population of patients with asthma provides a unique perspective of both patient-reported and EMR-recorded ICS-related adverse events. Data from both patient questionnaires and EMR demonstrated that the co-prescription of the unlicensed Aerochamber spacer with non-extrafine beclometasone was not associated with higher patient-reported or EMR-recorded adverse events than the co-prescription of the licensed Volumatic device with non-extrafine beclometasone.

The combination of the right medication and the optimal delivery device with the patient’s cognitive and physical abilities are essential to ensure optimum therapy delivery. The prescription of devices that are not easily used by patients can result in incorrect inhaler technique, leading to decreased drug delivery, poor disease control, and culminating in decreased therapy adherence.^37,38^ National and international guidelines offer advice on which patients should receive specific therapies.^1,26^ However, these are often not strictly followed by healthcare professionals as patients may be unwilling to carry, or unable to use the device. Off-label and unlicensed prescriptions may also be given due to the lack of a licensed therapy available for a patient’s age group.^36^ Our recent study found that national guidelines for spacer prescription were not followed for a large proportion of patients prescribed non-EF BDP.^33^ Of those patients who were prescribed spacers, the majority were prescribed the unlicensed Aerochamber spacer (59.0%) followed by the licensed Volumatic device (18.9%).^33^

A major concern with unlicensed prescriptions is the potential for side effects. Several studies have reported an increased incidence of oral thrush in patients with asthma associated with ICS dose.^22–24,31^ To account for this, we adjusted our analysis for ICS dose in the questionnaire-based study and matched for ICS daily dose in the EMR-based study. Thus, any difference in ICS-related adverse events would not have been caused by the ICS dose. Other reported local side effects of ICS use include dysphonia, cough reflex, and pharyngitis. These are also considered to be an immediate cause of clinical discomfort, which in turn reduce patient adherence to therapy, possibly resulting in a decrease in asthma control.^7,11,12^ This study clearly demonstrated that this was not the case for co-prescription of the unlicensed Aerochamber spacer with non-EF BDP asthma therapy. In terms of patient-perceived occurrence of oral thrush and hoarseness, the Aerochamber was non-inferior to the licensed Volumatic spacer. This was further confirmed by doctor-recorded data (diagnostic read codes), where patients prescribed the Aerochamber spacer did not suffer significantly more ICS-related adverse events than those prescribed the Volumatic spacer.

It is very likely that the range and extent of ICS-related adverse events, as experienced by patients, are underestimated.^9,12,39^ The short duration of clinical trials and the stringent inclusion criteria often limit the quality and quantity of data on adverse events.^40,41^ Similarly, patient-perceived ICS-associated adverse events may not be detected during routine clinical practice as patients are often reluctant to discuss their concerns about medication with their physicians.^42^ Discordance and lack of patient–prescriber communication may cause patients to titrate their medication or self-medicate, reducing disease control.^12^ This existing disparity between doctors and patients, with respect to their approach to drug-related adverse events, can be tackled via better patient understanding of their treatment benefits and of potential adverse effects, and with prescribers trying to better understand the concerns of patients.^43^ Previous reports have suggested differences in patient- versus doctor-reported adverse events,^44^ and it is therefore important to integrate self-reported patient questionnaires as a key tool for investigating adverse events. The use of both patient questionnaires and physician-recorded adverse events makes this study unique.

Real-world studies assess the results of therapy under conditions of usual care that are not subjected to the selection of patients through restrictive eligibility criteria as occurs in clinical trials. Although the Optimum Patient Care Research Database (OPCRD) is a well-maintained and validated database, we cannot rule out the possibility of inaccurate or missing data. The outcomes were studied over 3 full years for the cohort study to balance seasonal influences on outcome measures. However, the real-life nature of this study also means that although spacer prescriptions were identified, it is not guaranteed that the prescriptions were filled or that the spacers were used. A limitation inherent to observational studies is the possibility of unrecognised confounding factors or influences in prescribing that were not accounted for such as inhaler technique. Lastly, only read-coded adverse events would have been detected in the EMR-based study. This is however unlikely to be unbalanced between either spacer arms and thus is not expected to significantly affect the finding of this study.

The current study focused on the adverse events of spacer co-prescription with non-extrafine beclometasone prescription for asthma. More recent devices are able to generate ICS aerosol as extrafine particles which has been previously reported by various studies to have comparable to superior efficacy and safety compared to non-extrafine formulation.^15,45^ Further studies will be required to extend the finding of this study to spacer use with ICS delivered as extrafine particles.

In conclusion, this study found that co-prescription of the unlicensed Aerochamber spacer with non-extrafine beclometasone dipropionate therapy for asthma did not increase the risk of developing patient-reported or EMR-recorded ICS-related adverse events, as compared to co-prescription of the licensed Volumatic device.

## Methods

### Data source

The study utilised a large UK primary care database, the OPCRD (www.opcrd.co.uk).^46^ The OPCRD currently comprises fully anonymous, longitudinal medical records for over 4.5 million patients from over 600 primary care practices across the United Kingdom. The OPCRD contains two types of data: (1) routinely recorded clinical data and (2) questionnaire data (collected as part of routine patient data collection) from over 55,700 patients with respiratory conditions. This enables real-life studies to draw on information from both perspectives, ensuring a more complete answer to the questions posed. The OPCRD is approved by the Health Research Authority of the UK NHS for clinical research use (Research Ethics Committee (REC) reference: 15/EM/0150). Records contain complete prescribing, coded diagnostic, and clinical information, as well as information on tests requested, laboratory results, and referrals made at or following on from each consultation.^47^

### Study design

This was a post-authorisation safety study utilising two separate historical study designs to achieve the objectives. The first was a historical study involving routine questionnaire data stored in the OPRCD to compare patient-reported ICS-related adverse events for asthma patients prescribed non-EF BDP with either a Volumatic or an Aerochamber spacer. This consisted of a 1-year baseline period for patient characterisation, concluding at the index date, defined as the date of return of the asthma questionnaire.

The second design was a historical EMR-based study to compare physician-recorded outcomes, composed of a 1-year baseline period for characterisation and matching, followed by a 3-year outcome period for detection of adverse events. The index date was defined as the date of first spacer prescription (Volumatic or Aerochamber).

The study protocol was overseen by an independent steering committee and registered with the European Network of Centers for Pharmacoepidemiology and Pharmacovigilance (trial registration number EUPAS13194) and the Anonymous Data Ethics Protocols and Transparency (ADEPT) committee (Ref: ADEPT0517) prior to data extraction.

### Patients

For the questionnaire-based study, eligible patients were aged ≤65 years, with a Read code (clinical coding system within UK’s primary care) confirmed asthma diagnosis. They received ≥2 separate non-EF BDP (Clenil Modulite) prescriptions with one prescription for a spacer (Volumatic or Aerochamber) during the baseline year prior to the date of the questionnaire and had 2 years of continuous practice data (comprising ≥1 year of data prior to the questionnaire).

Eligible patients of the EMR-based study were aged ≤65 years, with a Read code confirmed asthma diagnosis. They received ≥2 separate non-EF BDP (Clenil Modulite) prescriptions in the baseline year and another ≥2 prescriptions in 1 year after index. They were prescribed either Volumatic or Aerochamber spacer at the index date and had 4 years of continuous practice data (comprising ≥1 year of baseline data and 3 years of outcome data).

Patients were excluded from both studies if they received prescriptions for different ICS or fixed-dose combination) ICS/LABA (long-acting β-agonist) therapy or were ever prescribed both Volumatic and Aerochamber spacers (Supplementary Table 1, Supplementary Table 2, Figs. 1 and 2).

### Outcome measures

The primary objective was to determine non-inferiority of non-EF BDP co-prescribed with an Aerochamber spacer, compared to the Volumatic spacer, in terms of the frequency of patient-reported oral thrush or hoarseness via the asthma questionnaire over a single year. Other adverse events captured via the questionnaire included sore mouth/throat, bruising, abnormal weight gain, and cough.

The secondary objective was to compare EMR outcomes for non-EF BDP co-prescribed with either the Volumatic or the Aerochamber spacer in the EMR-based study. Read codes for adverse events to non-EF BDP, as defined in the product information sheet^32^ (oral thrush, adrenal suppression diagnosis, osteoporosis/osteopenia, anxiety/depression, cataracts, and glaucoma) over a 3-year period were extracted from the OPCRD.

### Adjustment and matching

For the questionnaire-based study, patients in each spacer group were compared following adjustment for variables selected from those with the highest relative change in coefficient. The final variables used for adjustment were gender, ICS average dose, and smoking status, selected based on clinical judgement and baseline balance. Matching was not conducted for the questionnaire-based study to preserve statistical power due to sample size.

For the EMR-based study, exact matching for categorical variables and matching within a maximum calliper for numeric variables were used to match patients using 1:1 nearest neighbour matching, without replacement. Matching variables such as demographic data, disease co-morbidity, and indicators of disease severity were considered for selection using a combination of baseline data analysis and predictive modelling of the baseline data in relation to the outcome variable (independent of treatment group). The final criteria settled on a mix of direct and propensity score matching (Supplementary Table 3).

### Statistical analysis

The study was powered using the occurrence of oral thrush as the representative adverse event. The occurrence of adverse events (34%) was based on the oropharyngeal adverse events in users of ICS in a real-life setting reported in the literature.^9,48^ With sample sizes of at least 293 and 147, a two-group large-sample normal approximation test of proportions with a one-sided 0.025 significance level would have 80% power to reject the null hypothesis that the test and the standard are not equivalent (the difference in proportions, pT−pS, is 0.130 or farther from zero in the same direction).

All analyses were carried out using IBM SPSS Statistics version 21 (IBM SPSS Statistics, Feltham, Middlesex, UK), SAS version 9.3 (SAS Institute, Marlow, Buckinghamshire, UK), and Microsoft Office Excel 2013 (Microsoft Corp., Redmond, Washington, USA). Forest Plot was generated with DistillerSR Forest Plot Generator from Evidence Partners.

MEE of spacer type (Aerochamber or Volumatic) on the reported oral thrush/hoarse voice incidence was calculated to determine non-inferiority in the primary outcome analysis. The MEE was calculated from predictions of the model at fixed values of the covariates and averaging over the remaining covariates to obtain an interval where the result was likely to lie. Chi-square test was utilised to obtain the odds ratio of adverse events identified from the EMR (EMR-recorder adverse events). Poisson regression was utilised to calculate the rate ratio of EMR-recorded adverse events, adjusted for osteoporosis and anxiety/depression diagnosis. The *p* value of <0.05 was considered statistically significant.

### Reporting summary

Further information on experimental design is available in the Nature Research Reporting Summary linked to this article.

## Supplementary information

Supplementary Information

Reporting Summary

## Data Availability

All relevant data are within the paper and the supporting information files. The dataset supporting the conclusions of this article was derived from the UK Optimum Patient Care Research Database (www.opcrd.co.uk). We do not have permission to give public access to these databases; however, researchers may request access for their own purposes. Request for access to OCPRD can be made via the OCPRD website (https://opcrd.co.uk/our-database/data-requests/) or via the enquiries email info@opcrd.co.uk. In accordance with the terms of the agreement signed by OPCRD and OPRI, datasets used in the study must be destroyed within 1 year of availability. The OPCRD has ethical approval from the National Health Service (NHS) Research Authority to hold and process anonymised research data (Research Ethics Committee reference: 15/EM/0150). This study was approved by the Anonymised Data Ethics Protocols and Transparency (ADEPT) committee—the independent scientific advisory committee for the OPCRD, commissioned by the Respiratory Effectiveness Group. The study was designed, implemented, and registered in accordance with the criteria of the European Network of Centres for Pharmacoepidemiology and Pharmacovigilance (ENCePP) (registration number: EUPAS13194).

## Supplementary information

**Supplementary information** accompanies the paper on the *npj Primary Care Respiratory Medicine* website (10.1038/s41533-019-0115-0).
