# Supplementary material for: Comparison of adverse events associated with different spacers used with non-extrafine beclometasone dipropionate for asthma
Source: NPJ Prim Care Respir Med. 2019 Feb 8;29:3. doi: 10.1038/s41533-019-0115-0 (PMC6368625; doi:10.1038/s41533-019-0115-0)
Supplement: Supplementary file 1 — Supplementary Information [file 41533_2019_115_MOESM1_ESM.pdf]

Supplementary Table 1: Inclusion and exclusion criteria for the questionnaire-based study

**INCLUSION CRITERIA**
Diagnosis for asthma (confirmed by diagnostic Read code)
Age ≤65 years at index date
Two years of continuous practice data comprising 1-year of data prior to questionnaire
Asthma questionnaire returned
Prescription of Aerochamber® or Volumatic® spacer prior to the questionnaire
≥2 separate prescriptions of non-extrafine beclometasone in the baseline year prior to the index date
**EXCLUSION CRITERIA**
Received multiple types of ICS or FDC ICS/LABA during the baseline period or at the index date
Prescribed both Aerochamber and Volumatic spacers ever

Supplementary Table 2: Inclusion and exclusion criteria for the EMR-based study

**INCLUSION CRITERIA**
Diagnosis for asthma (confirmed by diagnostic Read code)
Prescription of a Volumatic or Aerochamber spacer (index date)
Age ≤65 years at index date
4-years of continuous practice data comprising 1-year baseline data and 3-year outcome data
≥2 separate prescriptions of the beclometasone pMDI in baseline year and ≥2 prescriptions in one year after index prescription date
**EXCLUSION CRITERIA**
Received multiple types of ICS or FDC ICS/LABA during the baseline period, at the index date or during the outcome period
Prescribed both Aerochamber and Volumatic spacers

Supplementary Table 3: Matching criteria for the EMR-based study

Variable	Calliper
**Age**	3 years
**Gender**	0
**ICS average daily dose category**	0
**Propensity score**	0.25x standard deviation
